# Supplementary material for: Analysis of the percentages of monocyte subsets and ILC2s, their relationships with metabolic variables and response to hypocaloric restriction in obesity
Source: PLoS One. 2020 Feb 19;15(2):e0228637. doi: 10.1371/journal.pone.0228637 (PMC7029876; doi:10.1371/journal.pone.0228637)
Supplement: Attachment — Submitted filename: 20 01 Rebuttal_Mo_REE.docx [file pone.0228637.s003.docx]

January 10^th^, 2019

**Additional Editor Comments:**

In addition to the points made by the reviewers, please add more details for your flow cytometry methodology. How did you set your gates? For example, were FMOs only used for ICL2s? The gating strategy needs more detail, and explaining the methods will help strengthen the manuscript.

***R = According to this observation, we have included this information in the corrected manuscript, in the Materials and Methods section (page 6, lines 130-135, and 139-143; page 7, lines 144-146 and 147-158), and figure legends (page 31, lines 513-516, 520-524, and 531-536).***

Why are all your cells in Figure 2 positive? It seems there should be cells that are not β2AR positive.

***R = According to this comment, we have added a new histogram of β2AR staining showing the percents of positive and negative cells, according to a negative (cells stained with an irrelevant isotype-matched mAb. In addition, and as stated in the corrected manuscript, results have been expressed as the MFI of positive cells. We used unstained cells tube and isotype controls tube in each experiment for assigning the gates and markers for negative and positive fluorescence. In the new Figure 2 we now included a histogram of PE-isotype control with its marker corresponding. Also in Figure Legend 2 included this information (page 31, lines 527-536).***

In addition, your monocyte gate is quite small, which is particularly worrisome since the voltage is such that there is not much “separation” between cell populations. (for future research studies, you may want to consider adjusting your voltages so that it is easier to distinguish between cell populations).

***R = We fully appreciate this recommendation. According to it, the dot plots and gates have been corrected, by using the Kaluza software. These figures have been included in the new version of the manuscript (Figs. 1 and 2).***

Please carefully go through your results and discussion sections and make the results more specific. As it is written right now, it is unclear which results are being compared. For example “No differences in β2AR expression by NCM (CD14-CD16++) and CM (CD14++CD16-) were observed between the groups of obese and non-obese subjects. Yet, the IM (CD14+CD16+) from obese subjects expressed significantly more β2AR (p<0.001) (Table 1 and Fig. 2).” These sections are missing which time points are being compared. This is a consistent problem throughout the results and discussion sections.

***R = We apologize by the confuse and improper writing of these paragraphs. According to this observation, these paragraphs have been corrected (page 8, lines 187-189).***

Much of the discussion session needs to be re-written. The current discussion section is written as a literature review, as there is very little discussion of the results of your study and your interpretation of what these findings mean. Although the discussion section generally avoids over-stating, it does need more work to discuss the findings of the research study.

***R = According to this observation, the Discussion section has been thoroughly revised, with a proper analysis of our findings.***

**Reviewers' comments:**
**Reviewer's Responses to Questions
Comments to the Author**

1. Is the manuscript technically sound, and do the data support the conclusions?

The manuscript must describe a technically sound piece of scientific research with data that supports the conclusions. Experiments must have been conducted rigorously, with appropriate controls, replication, and sample sizes. The conclusions must be drawn appropriately based on the data presented. 

Reviewer #1: Partly

***R = According to this observation, we included in the new version of manuscript a discussion more detailed about our findings.***

Reviewer #2: Yes

2. Has the statistical analysis been performed appropriately and rigorously? 

Reviewer #1: Yes

Reviewer #2: Yes

3. Have the authors made all data underlying the findings in their manuscript fully available?

The [PLOS Data policy](http://www.plosone.org/static/policies.action#sharing) requires authors to make all data underlying the findings described in their manuscript fully available without restriction, with rare exception (please refer to the Data Availability Statement in the manuscript PDF file). The data should be provided as part of the manuscript or its supporting information, or deposited to a public repository. For example, in addition to summary statistics, the data points behind means, medians and variance measures should be available. If there are restrictions on publicly sharing data—e.g. participant privacy or use of data from a third party—those must be specified.

Reviewer #1: Yes

Reviewer #2: Yes

4. Is the manuscript presented in an intelligible fashion and written in standard English?

PLOS ONE does not copyedit accepted manuscripts, so the language in submitted articles must be clear, correct, and unambiguous. Any typographical or grammatical errors should be corrected at revision, so please note any specific errors here.

Reviewer #1: Yes

Reviewer #2: No

***R = We have reviewed the grammar and syntax of the manuscript.***

5. Review Comments to the Author

Please use the space provided to explain your answers to the questions above. You may also include additional comments for the author, including concerns about dual publication, research ethics, or publication ethics. (Please upload your review as an attachment if it exceeds 20,000 characters)

**Reviewer #1:** I appreciate the opportunity to review this interesting paper on circulating monocytes and group 2 innate lymphoid cells (ILC2s) in lean and obese (obesity assessed by BMI) subjects, and the possible effect of hypocaloric restriction on these cells. The premise of this study is the hypothesis that in obese subjects non-classical monocytes subpopulation is increased and ILC2s are decreased comparing to lean controls and that the weight loss induced by hypocaloric restriction hypocaloric would reverse the situation. Authors has tested this hypothesis on 74 lean and 65 obese individuals, who were subject to diet restrictions for 7-weeks. They observed that In obese individuals the percent of non-classical monocytes (NCM), and the expression of the β2-adrenergic receptor (β2AR) expression by intermediate monocytes (IM) were increased, whereas the percent of ILC2s was decreased. Caloric restriction lead to a decrease of NCM and the β2AR expression by IM, as well as, an increase in the percent of classical monocytes and ILC2s. Authors concluded that the weight loss induced by hypocaloric restriction is significantly associated with changes of monocytes and ILC2s percentages, which may contribute to the attenuation of the low-grade inflammation linked to obesity.
The main strengths of this paper is that it addresses an interesting and timely problem, the article is well constructed, the experiments were well conducted, and analysis was well performed. Recently, ILC2s were identified in murine and human adipose tissue and have been shown to promote the white adipose tissue beiging and prevent the development of obesity
Considering these strengths, I feel some concerns on the manuscript, which should be addressed before being accepted for the publication.

**Major comments:**
• The research question is not clear from the introduction. Authors should more convincingly justify why they decided to measure subpopulations of monocytes and ILC2. Why do the authors think that it is important to measure circulating ILC2s in the blood when their action appears to be mainly local, in adipose tissue? Please discuss. Authors should also justify in the introduction why decide to measure leptin and ghrelin.

***R = According to this observation, we have added relevant information regarding adipokines (page 3, lines 51-58), and immune cells (page 4 lines 66-69, 78-80, and 86-88) in the Introduction as well as in the Discussion (page 11, lines 238-240, and 255-259; and page 12, lines 260-261). We hope that this reviewer detects now more convincing information in the corrected manuscript that justifies the analysis of the different immune and metabolic parameters detected in our study.***

• Reliance on BMI as a sole marker of obesity, seems to be the serious limitation of the study. A number of authors indicated a poor linear relationship between BMI and total body fat and also suggested that body fat distribution would be more clinically significant than overall obesity. For instance using dual energy x-ray absorptiometry (DXA) seems to be fast and relatively inexpensive method to assess visceral adipose tissue.

***R = We agree that BMI has a poor linear relationship with total body fat. However, we consider that BMI is still a useful parameter for the diagnosis of obesity. In addition, unfortunately, in our University we do not have access to DXA.***

• A flow chart showing the experimental procedure should be included as it makes the experimental setting more visible to the reader. It isn’t clear for me what were the effects of caloric restriction in non-obese participants?

***R = According to this observation, a flow chart accounting for the recruitment of subjects and experimental procedures has been included in the corrected manuscript (page 33). Moreover, the caloric restriction diet was prescribed only for obese individuals, as stated in the Materials and Methods section of the corrected manuscript (page 5, lines 100-101, and 111-112, and page 6, lines 119-120).***

• Conclusions are too general.

***R = We are offering a new version of conclusions, with a proper analysis of our findings.***

**Minor comments:**
• How exactly participants were selected for the study? I assume that individuals with BMI ≥24.9kg/m2 and ≤30 (kg/m2 were excluded from the study. Authors should clearly state it. In parasite infections and allergies, circulating ILC2s could be elevated. How did authors ensure exclusion such participants?

***R = According to this observation, a more detailed description of the inclusion criteria for the recruitment of individuals has been added to the corrected manuscript, including the new flow chart (page 33). Moreover, it is worth mentioning that subjects with overweight were not included in the study (*≥24.9 kg/m^2^ and ≤30 kg/m^2^), *and that those subjects with atopy, allergic symptoms or with parasite infections (according to their medical records) were also excluded from the study.***

• Figures are confusing and the figure legends do not provide sufficient information to describe the data.

***R = We re-edited the figures 1, 2 and 3, called now as New Fig. 1, New Fig. 2 and New Fig. 3. The information was added in M&M (page 6, lines 130-135, and 139-143; page 7, lines 146-147 and 150-159), and figure legends (page 312, lines 514-517, 520-525, and 528-537).***

**Reviewer #2.**

The purpose of the current study was to assess monocyte subsets and ILC2s in lean and obese subjects, and the possible effect of energy restriction on these innate cells. The authors found that the percentage of non-classical monocytes and the expression of the Beta-2AR by intermediate monocytes were increased. In contrast the percentage of ILC2s was decreased in subjects with obesity. The authors also showed that there were significant negative associations between ILC2 and caloric intake, and Beta-2AR expression by intermediate monocytes and resting energy expenditure, but a positive relationship between non-classical monocytes and insulin resistance. Caloric restriction reduced non-classical monocytes, and Beta-2AR expression by intermediate monocytes. Classical monocyte and ILC2 populations increased with caloric restriction as well. These results suggest that weight loss may contribute to the reduction in low-grade inflammation in part by increasing ILC2 cell numbers and modifying the inflammatory characteristics of circulating monocytes. Overall, these results are novel and interesting but there were numerous grammatical errors (too many to list) throughout the manuscript that should be corrected.

***R =* We apologize for the numerous typos and grammar mistakes of the original manuscript. According to this observation, the manuscript has been thoroughly revised and we hope that this reviewer find a significant improvement in the grammar and syntax of the corrected manuscript.**

6. PLOS authors have the option to publish the peer review history of their article ([what does this mean?](https://journals.plos.org/plosone/s/editorial-and-peer-review-process#loc-peer-review-history)). If published, this will include your full peer review and any attached files.

If you choose “no”, your identity will remain anonymous but your review may still be made public.

**Do you want your identity to be public for this peer review?** For information about this choice, including consent withdrawal, please see our [Privacy Policy](https://www.plos.org/privacy-policy).

Reviewer #1: No

Reviewer #2: No

[NOTE: If reviewer comments were submitted as an attachment file, they will be attached to this email and accessible via the submission site. Please log into your account, locate the manuscript record, and check for the action link "View Attachments". If this link does not appear, there are no attachment files to be viewed.]

While revising your submission, please upload your figure files to the Preflight Analysis and Conversion Engine (PACE) digital diagnostic tool, <https://pacev2.apexcovantage.com/>. PACE helps ensure that figures meet PLOS requirements. To use PACE, you must first register as a user. Registration is free. Then, login and navigate to the UPLOAD tab, where you will find detailed instructions on how to use the tool. If you encounter any issues or have any questions when using PACE, please email us at [figures@plos.org](mailto:figures@plos.org). Please note that Supporting Information files do not need this step.
